# Improving Hybrid CTC/Attention Architecture for Agglutinative Language Speech Recognition

**DOI:** 10.3390/s22197319

**Published:** 2022-09-27

**Authors:** Zeyu Ren, Nurmemet Yolwas, Wushour Slamu, Ronghe Cao, Huiru Wang

**Affiliations:** 1 Xinjiang Multilingual Information Technology Laboratory, Xinjiang Multilingual Information Technology Research Center, College of Information Science and Engineering, Xinjiang University, Urumqi 830017, China; 2 College of Information Science and Engineering, Xinjiang University, Urumqi 830017, China

**Keywords:** agglutinative language speech recognition, hybrid CTC/attention architecture, low-resource, data augmentation, MSPC

## Abstract

Unlike the traditional model, the end-to-end (E2E) ASR model does not require speech information such as a pronunciation dictionary, and its system is built through a single neural network and obtains performance comparable to that of traditional methods. However, the model requires massive amounts of training data. Recently, hybrid CTC/attention ASR systems have become more popular and have achieved good performance even under low-resource conditions, but they are rarely used in Central Asian languages such as Turkish and Uzbek. We extend the dataset by adding noise to the original audio and using speed perturbation. To develop the performance of an E2E agglutinative language speech recognition system, we propose a new feature extractor, MSPC, which uses different sizes of convolution kernels to extract and fuse features of different scales. The experimental results show that this structure is superior to VGGnet. In addition to this, the attention module is improved. By using the CTC objective function in training and the BERT model to initialize the language model in the decoding stage, the proposed method accelerates the convergence of the model and improves the accuracy of speech recognition. Compared with the baseline model, the character error rate (CER) and word error rate (WER) on the LibriSpeech test-other dataset increases by 2.42% and 2.96%, respectively. We apply the model structure to the Common Voice—Turkish (35 h) and Uzbek (78 h) datasets, and the WER is reduced by 7.07% and 7.08%, respectively. The results show that our method is close to the advanced E2E systems.

## 1. Introduction

In recent years, E2E speech recognition models have been widely used in the field of automatic speech recognition [1,2,3,4]. The E2E approach achieves performance comparable to traditional systems by using a single E2E model instead of separate components [5]. Unlike the traditional speech recognition model, the E2E model has a simpler structure. By constructing a neural network, the same function as that of the traditional model can be achieved, and joint optimization can be achieved. The E2E models mainly include the Listen-Attend-Spell (LAS) model, which is also called the attention-based encoder–decoder (AED) model [6], connectionist temporal classification (CTC) model or recurrent neural network transducer (RNN-T) model [7]. CTC uses the method of adding a blank tag, <blank>, to the output sequence in order to align the speech frame sequence and the text sequence so that the training process can be simplified. RNN-T is improved based on CTC, which augments the CTC encoder with a recurrent neural network language model (LM). Compared with CTC, RNN-T is no longer limited in the length of its input and output sequences, but it is more challenging to train. AED, another of the most commonly used structures, includes an encoder module for feature extraction and a decoder module using attention mechanism. This architecture can use various types of neural networks, such as the convolutional neural network (RNN) [8], Gate Recurrent Unit (GRU) [9] and transformer [10]. The attention mechanism in AED pays attention to the complete sequence. Still, it is impossible to achieve strong alignment between audio signals and text labels due to the monotonicity between them. To overcome the above problem, a hybrid CTC/Attention model was proposed by Watanabe [11]. The key of the CTC/Attention model is to train the shared encoder with both the CTC and attention decoder as objective functions [12]. This training strategy dramatically improves the convergence of attention-based models and reduces the alignment problem, so it has become the standard training recipe for most AED models [13,14]. It effectively combines the advantages of the two frameworks and achieves similar results to the traditional ASR system based on a simple structure.

Low-resource languages comprise a large percentage of world languages, as 94% of languages are spoken by less than one million people [15]. Although technology giants such as Google, Apple, Facebook, Microsoft, Amazon and IBM have built advanced speech recognition engines for English, European and Asian languages, research on ASR systems for most Central Asian languages such as Uzbek is still in its infancy [16]. The reason for this status is the lack of standard corpora and the existence of dialect differences [17]. Although a series of studies have been carried out under the condition of a lack of resources [18,19,20], the challenge of poor generalization ability and high error rate due to the scarcity of resources still exists under the condition of low resources. Most researchers improve the resource scarcity problem through multilingual training and data augmentation. Multilingual learning can alleviate the problem of insufficient resources by acquiring language information from rich languages or similar languages [21]. At the same time, another simple and effective method is transfer learning, which first trains the network on a resource-rich corpus, and then, only fine-tunes it. E2E speech recognition technology does not rely on linguistic knowledge but only needs speech and its corresponding pronunciation text. The recognition effect depends on the size of the corpus to a certain extent. Other languages such as CSJ [22], Switchboard [23], ksponspeech [24], etc. have thousands of hours of training data. Uzbek and Turkish, belonging to the Galoric and Oguz branches of the Altaic language family, respectively, are agglutinative languages without a large amount of training data. Furthermore, the linguistic studies of these two Central Asian languages are incomplete. Due to the lack of relevant professional knowledge, it is very difficult to organize pronunciation dictionaries and define phoneme sets, and it is impossible to use traditional methods such as HMM/DNN to recognize them.

There are two main challenges in building an ASR system for these two languages: First, these two languages, as low-resource languages, not only have fewer corpus resources but also belong to agglutinative languages. By concatenating different suffix sequences, new word forms can be derived from a single stem, including many words with the same stem but different endings. This leads to its rich vocabulary, which increases the difficulty in speech recognition. Secondly, there are dialect differences in the same language, and there are labeled data with dialect differences in existing datasets. Additionally, since there is a lack of a corresponding pronunciation dictionary, our models operate on character units that are created via sentencepiece [25]. In addition, adding a CTC model during E2E model training has been proven to improve system performance effectively, but there has not been much research on agglutinative language speech recognition based on hybrid CTC/attention architecture.

To address these issues, we propose an ASR system based on hybrid CTC/attention architecture for Turkish and Uzbek. In our work, we use the speechbrain [26] speech recognition toolkit to build models. The main contributions of this paper are as follows:

1. We propose a feature extractor called Multi-Scale Parallel Convolution (MSPC) and combine it with bidirectional long short-term memory (Bi-LSTM) to form an encoder structure to improve the recognition rate and system robustness of the end-to-end model.
2. The location-aware attention is improved to consider the impact of attention weight history on attention.
3. By arranging and combining a variety of data augment methods, we achieve the best model training effect.
4. In the decoding stage, an RNN language model is added and initialized using a fine-tuned pre-trained BERT [27].

The rest of this paper is organized as follows. In Section 2, we briefly introduce the related work and particularly describe the development of hybrid CTC/attention systems and their application in low-resource speech recognition. In Section 3, the proposed method is introduced in detail. Section 4 presents experiments using our improved attention, MSPC and data augment scheme compared to the state-of-the-art (SOTA) hybrid CTC/attention E2E ASR model [28]. In Section 5, we summarize the work we have conducted and put forward the prospects.

## 2. Related Work

The E2E speech recognition model unifies traditional acoustic, pronunciation, and language models into a single acoustic model. It not only reduces the complexity of speech recognition modeling but also performs better than traditional models [29].

CTC is the first E2E model structure widely used in ASR [1,30,31]. In [32], CTC was proposed for the first time and was used for a speech recognition task in 2013. Unlike the HMM structure, it automatically learns and optimizes the correspondence between audio information and annotated text during training, and does not need to achieve frame alignment before network training. The disadvantage is that it assumes that each tag is independent of the others, but in reality, there is context dependency between tags at each moment. To solve this problem, RNN-T introduces a predictive network to learn context information, which is equivalent to the language model [33]. Another way to alleviate the condition-independent hypothesis is to use the attention mechanism [34], which is completed by improving the encoder structure without changing the objective function.

In contrast with CTC, AED does not require conditional independence assumptions and is another framework for E2E ASR models [6]. The structure consists of an encoder, a decoder and an attention module. The encoder and decoder are built using recurrent neural networks and an attention module to achieve soft alignment between labels and audio information. However, the AED model has poor generalization ability for long audio segments [35]. The inconsistent lengths of input and output sequences increase the difficulty in alignment. For long audio, it is necessary to manually set a window to limit the exploration range of attention. Secondly, the alignment in the attention mechanism can be easily destroyed by noise.

In order to solve the alignment problem in AED, Kim S et al. proposed hybrid CTC/attention architecture [36]. The attention and CTC models are optimized by sharing encoders within the multi-task learning framework, and the convergence of the model is accelerated while correcting the alignment problem [13]. The attention-based sequence-to-sequence network is trained using CTC as an auxiliary objective function during training. The forward–backward algorithm of CTC can enforce a monotonic alignment between audio and label sequences. In such structures, attention mechanisms that are often employed are additive attention [37], location-aware attention [38], scaled dot-product attention [39], etc. to perform attention operations on the entire input representation. In [40], the model is further improved by combining the scores from the AED model and the CTC model in both rescoring and one-pass decoding during the decoding process. Seki H et al. sped up the decoding process of the hybrid CTC/attention model by vectorizing multiple assumptions in beam search [41]. Then, various hybrid models were proposed to solve the alignment problem [42,43].

In addition, LM pre-training has become a common technology in the NLP field, and BERT is one of them, which uses a transformer to build a text encoder. Unlike BERT, GPT2 consists of multiple layers of unidirectional transformers that generate data representations through historical context [44]. Sanh V et al. proposed a knowledge distillation method to compress BERT into DistilBERT [45]. This method is also used in GPT2 model compression. Language models are widely used in ASR task [46]. Combining LM with an end-to-end ASR model is common through shallow fusion [47] or cold fusion [48]. Self-supervised pre-training models are widely used in end-to-end speech recognition tasks, but as the decoder is based on acoustic representation, it is impossible to carry out separate pre-training. Recently, some research has been conducted to integrate BERT into the ASR model [49]. In [50], K Deng et al. initialize the encoder using wav2vec2.0 [51], and the decoder through a pre-trained LM DistilGPT2, to take full advantage of the pre-trained acoustic and language models.

The end-to-end models described above have been widely used in various languages, but only a few have been applied to Central Asian languages. Dalmia S et al. first tried to use the CTC framework to build a multilingual ASR system for low-resource languages including Turkish [20]. Mamyrbayev O et al. used different types of neural networks, a CTC model and attention-based encoder–decoder models for E2E speech recognition in agglutinative languages, achieving good results without integrating language models [52]. Cheng Yi et al. fusd a pre-trained acoustic encoder (wav2vec2.0) and a pre-trained linguistic encoder (BERT) into an end-to-end ASR model [53]; the fusion model only needed to be fine-tuned on a limited dataset. Orken Zh et al. proposed a joint model based on CTC and the attention mechanism for recognition of Kazakh speech in noisy conditions [54]. In addition to the improvement of the model structure, some important technologies are often applied to low-resource speech recognition, which is also the key to improving performance [55]. The most widespread application for these is data augmentation, a technology for increasing the amount of data needed for training speech recognition systems. Common data augmentation methods include specaugment [56], speed perturbation [57] and multilingual processing. There are also some recently adopted data augmentation methods. For example, in [58], the ASR system receives pre-synthesized speech from Tacotron for out-of-domain data augmentation. Another popular method used on limited datasets is transfer learning [59], that is, using a small amount of data to retrain the basic acoustic model that is trained from other resource-rich speech databases. Cho J et al. attempted to use data from 10 BABEL languages to build a multilingual seq2seq model as a prior model, and then, port them towards four other BABEL languages using a transfer learning approach [60]. In addition, the construction of multilingual models has also become a new direction in attempting to solve the problem of the lack of corpus resources. Yi J et al. proposed adversarial multilingual training to train bottleneck (BN) networks for the target language, ensuring that the shared layers can extract language-invariant features [61]. With the multilingual CTC/attention model proposed by Liang S, Yan W used the optimal solution to complete the model evaluation and achieved similar performance to the traditional model, which provided a research basis for future exploration of speech recognition in different languages [62].

## 3. Materials and Methods

In this section, we mainly introduce our proposed hybrid CTC/attention network for the Uzbek and Turkish end-to-end model in speech recognition.

### 3.1. Encoder with Deep CNN

**Figure 1:**
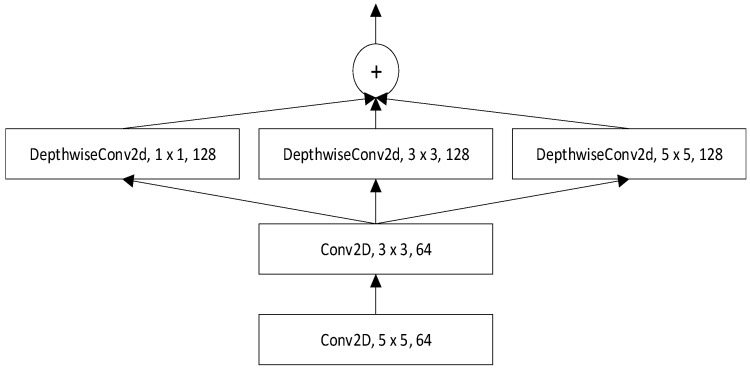
Illustration of the proposed MSPC architecture. The size of the convolution kernel and the number of output channels are introduced in detail.

**Figure 2:**
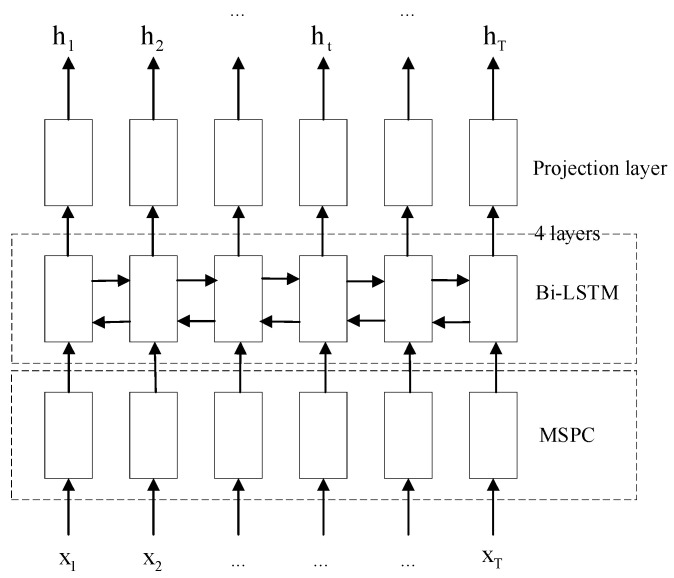
The architecture of our proposed encoder. The encoder transforms the input sequence $X_{1},\;X_{2},\;\ldots ,\;X_{T}$ into the corresponding hidden state $h_{1},\;h_{2},\;\ldots ,\;h_{T}$. Among them, the input sequence is a three-dimensional matrix (batch, time, feats).

We propose a new deep CNN Multi-Scale Parallel Convolution (MSPC), which consists of 2 convolutional layers, 1 pooling layer and a set of parallel convolutional layers, as shown in Figure 1. When we set the dimension of the convolution kernel, we consider the characteristics of the speech signal and that too large a convolution kernel is not conducive to increasing the depth of the network, which easily leads to excessive computation. Therefore, in parallel convolution, our convolution kernel selects 1, 3, and 5 to extract the features of different dimensions and perform a concat operation. At the end of the MSPC network, we access a maximum pooling layer to reduce the model parameters, thereby speeding up the training speed. Followed by Bi-LSTM, the context information in the speech signal is learned through this network, and the recognition rate of the end-to-end model is further improved. The specific encoder structure $f_{enc}$ is shown in Figure 2, which contains MSPC, followed by 4 layers of Bi-LSTM combined with a fully connected layer of projection neurons denoted by Lin(⋅). We use X to denote an input sequence that consists of feature vectors. The encoder is expressed as Equation (1):

(1) $$\;h_{t}=f_{enc}\left(X\right)=\left[Lin\left(\mathrm{Bi}\text{-}{\mathrm{LSTM}}^{4\times }\right)\right]\left(\mathrm{MSPC}\left(X\right)\right)$$

### 3.2. Connectionist Temporal Classification (CTC)

**Figure 3:**
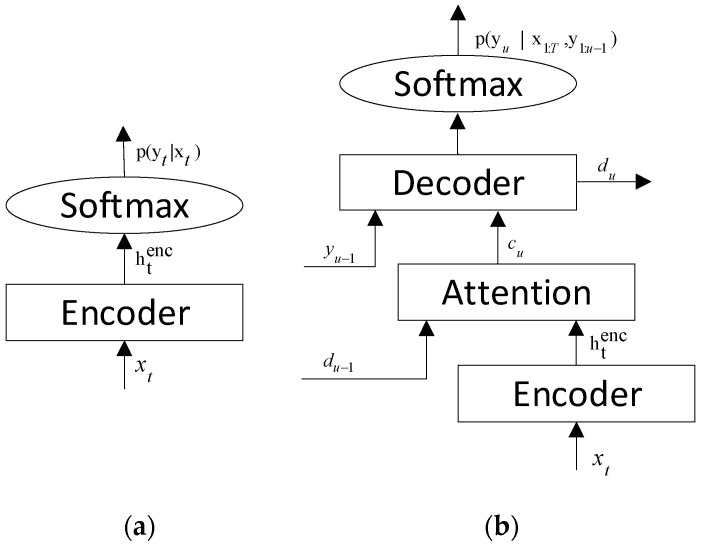
The structure diagram of the two main end-to-end models. (**a**) The architecture of connectionist temporal classification (CTC); (**b**) the architecture of attention-based encoder–decoder (AED) model.

CTC showed, in Figure 3a, that each input $x_{t}$ corresponds to an output. There may be many frames that generate only one pronunciation sequence. To solve this problem, blank symbols are added to determine the boundaries of the output. The basic Equation of CTC is as in Equation (2).

(2) $$y^{*}=argmax_{y}P\left(y|h_{t}^{enc}\right)$$

$y^{*}$ is the closest to the y for the encoder output $h_{t}^{enc}$. $P\left(y|h_{t}^{enc}\right)$ is calculated as shown in Equation (3) to find the output sequence.

(3) $$P\left(y|h_{t}^{enc}\right)\approx \overset{T}{\underset{t=1}{\prod }}p\left(y_{t}|x_{t}\right)=\overset{T}{\underset{t=1}{\prod }}q_{t}\left(y_{t}\right)$$

where T is the length of the speech sequence. At time t, $q_{t}\left(y_{t}\right)$ is the softmax activation of $y_{t}$ in the encoder layer q. The CTC loss function is defined as the negative log probabilities of the correct labels for a given input speech sequence, which is calculated using the forward-and-backward algorithm:

(4) $$loss_{ctc}=-lnP\left(y|x\right)$$

**Figure 4:**
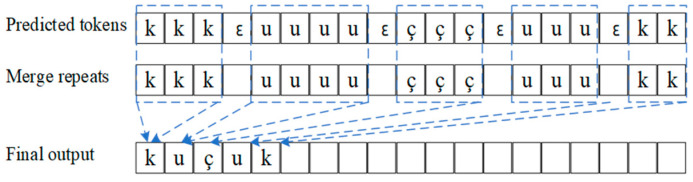
The CTC networks for speech recognition. ε represents a blank symbol.

Figure 4 shows the CTC algorithm, which is divided into two steps. The first step is to predict and output a series of characters; The second step is alignment, deleting blank symbols and merging duplicate characters. Details about the CTC algorithm can be found in Reference [63].

### 3.3. Attention-Based Encoder–Decoder

**Figure 5:**
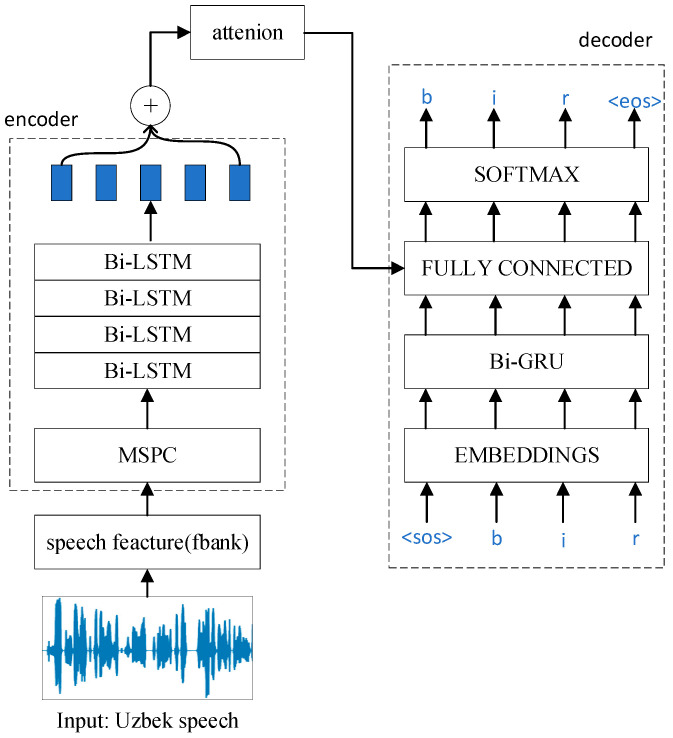
Encoder–decoder with attention model architecture for ASR. The model of input is the Uzbek speech utterance bir. <SOS> and <EOS> represent the start and the end of a sentence, respectively.

Unlike the CTC method, the attention-based encoder–decoder model (AED) directly predicts each target word without a conditional independent hypothesis. It contains two different networks: the encoder network converts the input feature x into a hidden vector h, and the decoder network converts h into the output label y. Figure 3b describes the structure of AED, and the encoder network performs the same function in the CTC. The AED model calculates the posterior probability as:

(5) $$P\left(y|x\right)=\underset{u}{\prod }P\left(y_{u}|x,y_{1:u-1}\right)$$

where u is the length of the output label. The attention module calculates the attention weights between the previous decoder hidden state $d_{u-1}$ and encoder output $h_{1:T}^{enc}$ for each frame using an attention function such as additive attention or dot product attention [64]. When calculating the attention weights $\;a_{ut}$, we take into account the impact of previous attention weight history $a_{1:u-1}$ on the results, as shown in Equation (7). Additionally, a context vector $c_{u}$ is then generated as the weighted sum of the encoder outputs, as Equation (8) shows. Input $c_{u}$, the decoded output $y_{u-1}$ at the previous moment, and the hidden state $d_{u-1}$ of the decoder at the last moment are entered into the decoder to obtain the hidden state $d_{u}$ of the decoder at the current moment. The previously obtained output label $y_{u-1}$ and context vector $c_{u}$ are entered together into the decoder to calculate $P\left(y_{u}|x,y_{1:u-1}\right)$. The specific calculation process is as follows:

(6) $$h_{t}^{enc}=Encoder\left(x_{t}\right)\;$$

(7) $$a_{ut}=Attend\left(d_{u-1},a_{1:u-1},\;h_{t}^{enc}\right)$$

(8) $$c_{u}=\overset{T}{\underset{t=1}{\sum }}a_{ut}h_{t}^{enc}$$

(9) $$d_{u}=Recurrency\left(d_{u-1},c_{u},\;y_{u-1}\right)$$

(10) $$P\left(y_{u}|x,y_{1:u-1}\right)=Decoder\left(y_{u-1},d_{u-1},c_{u}\right)$$

where $a_{ut}$ is an attention weight, which is computed by a feedforward network. Encoder(·) and Decoder(·) are RNN networks. The loss function of the model is obtained using Equation (11):

(11) $$loss_{AED}≜-lnP\left(y^{*}|x\right)=-\underset{u}{\sum }\ln P\left(y_{u}^{*}|x,y_{1:u-1}^{*}\right)$$

where $y_{1:u-1}^{*}$ is the ground truth of the previous characters. The specific model structure diagram is shown in Figure 5. Our major improvements include proposing a new CNN-based MSPC network and adding LSTM after the convolutional layer in location-aware attention. The specific structure of MSPC is shown in Figure 1.

### 3.4. Hybrid CTC/Attention Architecture with RNN-LM

To use the advantages of the above two models and address the irregular alignments problem in the attention mechanism, the loss function of the hybrid model [36] is calculated using Formula (12):

(12) $$loss_{hybrid}=\lambda logP_{ctc}\left(C|X\right)+\left(1-\lambda \right)logP_{att}\left(C|X\right)\;$$

where *λ* is a non-trainable parameter that ranges between 0 and 1. When decoding, the coverage penalty parameter is added [65], the decoder is made to pay attention to each token of the input sequence x evenly, and some tokens are prevented from being given too much attention. Another approach is to add a language model [28], which is incorporated into the decoding objective in (13):

(13) $$y^{*}=\underset{y}{\mathrm{argmax}}\log p\left(y|x\right)+\beta \log p_{LM}\left(y\right)+\gamma \mathrm{coverage}$$

**Figure 6:**
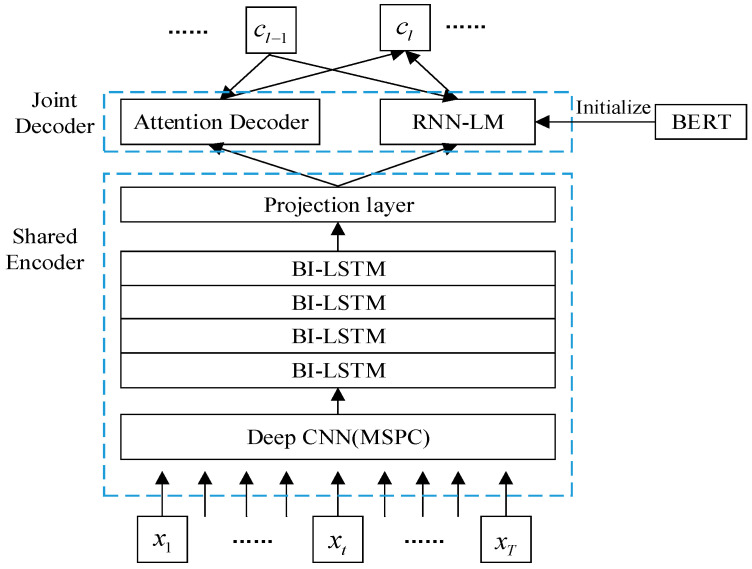
Illustration of the end-to-end framework based on hybrid CTC/attention that we use. The shared encoder is simultaneously trained by the CTC and the attention model objectives and mainly transforms the input sequence x into a high-level representation h. The joint decoder predicts an output label sequence through the attention decoder and RNN-LM. Context vector $c_{l}$ is calculated using Equation (8).

Equation (13) is a heuristic involving the multiplication of conditional and unconditional probabilities of the transcript y. Here, we set β to 0.5 and γ to 1.5. In our speech recognition system, characters are chosen as the model units, which require less sequence context information than subword-based units and improve the speed of model training. The overall architecture can be found in Figure 6.

## 4. Experiments

We used the speechbrain [26] toolkit to build the proposed model, using the word error rate (WER) and character error rate (CER) as the primary evaluation metrics. Our studies were conducted on high-performance computing (HPC) nodes equipped with one NVIDIA TITAN RTX GPU with 24 GB of RAM and an i7-7800X CPU.

### 4.1. Data Preparation

The Common Voice dataset is a multilingual public dataset containing more than 15,000 h of 96 languages. Each dataset entry consists of an individual MP3 audio file and corresponding text file. The Turkish and Uzbek corpuses used in our research were collected and validated via Mozilla’s Common Voice initiative [66]. Using either the Common Voice website or the iPhone app, contributors record their voices by reading sentences displayed on the screen. In addition, to demonstrate the effectiveness of our proposed model, we also carried out experiments on the LibriSpeech [67] dataset. All speeches were sampled at 16 kHz. Table 1 presents the details of the three datasets.

Eighty-dimensional log-Mel filterbank features were extracted for speech frames with a 25 ms length and 10 ms shift. To solve the problem of training over-fitting when the training set is not extensive enough, we used a linear combination of multiple data augmentation methods to achieve the best results.

### 4.2. Experimental Setup

We first trained and tested our implementation over the LibriSpeech dataset. Specifically, we used train-clean-100 as our training set and dev-clean/dev-other as our validation set. For evaluation, we reported the CER and WER on the subsets test-clean and test-other. Then, we also evaluated the other two sets. All experiments were implemented using speechbrain with the default configurations. The detailed experimental configuration is shown in Table 2. ‘blstmp’ means that the encoder is the projected bidirectional long short-term memory neural network. “VGG+4blstmp” means that an encoder is composed of a vaccination guidelines group (VGG) [68] layer followed by four blstmp layers. Location-lstm is the attention mechanism that we modified. Bi-GRU represents a bidirectional gated recurrent unit. We enhanced the training data using MUSAN [69] and RIRs [70] as noise sources to double the amount of training data. The experiment details are shown in Section 4.3 and Section 4.4.

### 4.3. The Results of Comparative Experiments

We trained all the models in the train-clean-100 subset and tested the test-clean and test-other subsets. The experimental results are shown in Table 3. Among them, the semi-supervised method and Ensemble (five models) method were trained with pseudo-labels, using 100 h paired data and 360 h unpaired audio.

From Table 3, we can see that our model obtains better performance in the CER and WER. We only changed the structure of the model from the baseline. Our model performs worse than the baseline using the content-based method on the test-clean subset. Although the recognition results using context-based attention in the model are more accurate than location-aware attention, the improvement is limited to the test-other dataset. Test-other, as the part of the speech recognition task with a high word error rate, has a higher WER. Our model has a better recognition effect and better robustness. Compared with the baseline, the WER on the two subsets is reduced by 0.35% and 2.66%, respectively. Compared with the other supervised methods, except for the Ensemble method, our model obtains the optimal performance. We guess that this is because the Ensemble method combines multiple models during training to increase label diversity and prevent models from being overconfident about noisy pseudo-labels. The second possible reason is that the method used additional 360 h unpaired audio for model training, which improved the model’s generalization.

Table 4 shows that our method achieves overall performance improvement in all cases. When decoding without RNNLM, our model acquires 0.66% and 4.17% relative CER and WER reductions compared with the baseline in the Turkish dataset. Moreover, we achieve relative CER and WER reductions of 5.82% and 7.07% with pretrained RNNLM.

For Uzbek, our model also improves in the main performance indicators. Because the corpus size is larger than that of Turkish, the convergence speed of the model is obviously faster, and the number of training rounds is less than that of the former. In particular, the relative WER is reduced by 6.53% and 7.08% when decoding with and without the RNNLM.

**Figure 7:**
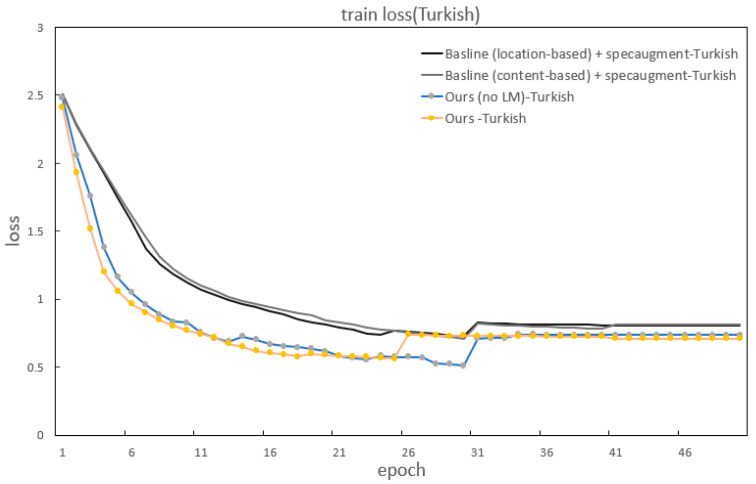
The train loss of the baseline and our methods for Turkish.

**Figure 8:**
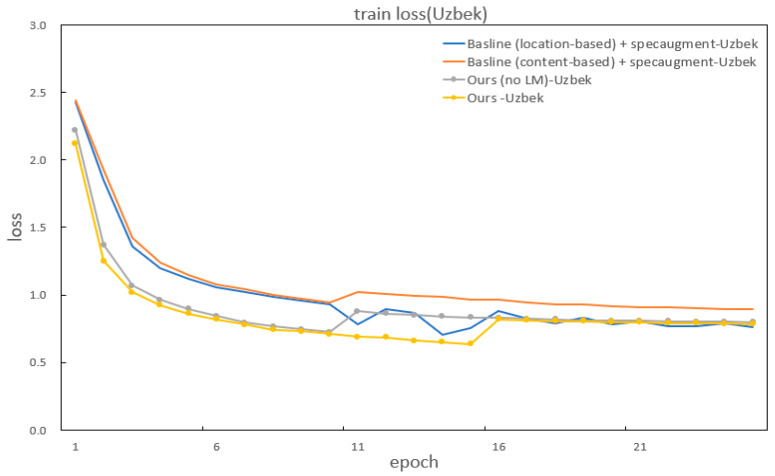
The train loss of the baseline and our methods for Uzbek.

In addition to improvements in the CER and WER, our method also significantly outperforms the baselines in accelerating model convergence. Figure 7 and Figure 8 show the specific details of the convergence in model training. It can be seen that our model has the fastest convergence and the shortest loss curve compared to the baseline models using two kinds of attention.

### 4.4. The Results of Ablation Experiments

In this subsection, we further validate the effectiveness of our proposed MSPC structure and improved location-aware attention module on the Common Voice—Turkish corpus and explore the performance of different data enhancement methods.

To determine the effectiveness of each module, we added the single modules to the experiment separately. Table 5 shows the performance of location-LSTM attention. From Table 5, it is not difficult to find that compared with location-aware attention and context-based attention, while the CER of the model after adding location-LSTM attention improves, the WER performance is basically the same. The performance of CER shows that it can effectively capture the local dependencies of speech frames. Although location-LSTM attention improves the performance to a lesser extent, it proves the effectiveness of the improved attention.

According to Table 6, after we use the proposed MSPC as the feature extractor, the WER is improved by 1.25%. This justifies that using MSPC instead of VGG as the feature extractor is reasonable. When we add two modules to the model simultaneously, our method improves the relative character error rate and relative word error rate by 0.75% and 1.73%, respectively, compared to the baseline. This proves that the network can extract features of different scales and learn the context information of speech signals, thus improving the recognition rate of end-to-end models.

To further explore the effect of combining different data augmentation methods on the experimental results. The noise method uses the noise dataset provided by [70] to add noise to the original audio. After analyzing the experimental results in Table 7, we found that using speed perturbation and the noisy method at the same time has advantages in terms of word error rate compared with using specaugment and the noisy method simultaneously. In terms of training speed, the former is also slightly better. Therefore, we take speed perturbation + noise as our final data augmentation scheme.

### 4.5. Effectiveness of Hyperparameters

In the experiments described in the previous two sections, when using beam search decoding, the beam width is set to 8. In this section, we explore the effect of different beam widths on the final result. As seen in Table 8, WER gradually decreases with the increase in the set beam width during decoding. When we use different widths, there is a definite improvement in system performance. The reason for this phenomenon is that the larger the width, the more choices we have to consider, and the better the sentence is likely to be. We did not choose a larger width, because it would increase the computational cost of the algorithm and greatly affect the decoding speed.

The RNNLM used in the experiments is initialized using the DistilBERTurk^1^ model. DistilBERTurk was trained on 7 GB of the original training data that were used for training BERTurk, using the cased version of BERTurk as a teacher model. When the beam width is 8, CER and WER are reduced by about 5% and 3%, respectively, after adding the language model. However, the relative improvement decreases further when the beam width increases to 16. This proves that the beam width has some effect on the results, and the gap is further narrowed when the language model is added.

## 5. Conclusions and Future Work

In this paper, we studied the application of the hybrid model in agglutinative language speech recognition and proposed a CNN-based feature extractor, MSPC, that uses different convolution kernel sizes to extract features of different sizes. Then, by improving its location-aware attention, the impact of the attention weight history on the results is considered while focusing on the location information. The results of the experiments show that the constructed model performs better after adding the language model, which not only exceeds the baseline model but also shows better performance compared with the mainstream model.

In the future, we will improve the following aspects of the proposed model. The Altaic language family contains many agglutinative languages, which have a small corpus due to their small number of speakers. Thus, we will explore multilingual speech recognition based on this model so that our model can fully use multilingual information and improve the accuracy of low-resource speech recognition. Additionally, streaming speech recognition has gone mainstream. Therefore, we will further improve the model structure to suit the needs of streaming speech recognition.

## Figures and Tables

**Table 1:** Specifications of the LibriSpeech, Turkish and Uzbek corpus datasets.

Dataset	Duration (h)			Total Duration (h)	Total Speakers
	Train	Dev	Test		
LibriSpeech	100.6	Other/clean	Other/clean	121.8	397
		5.3/5.4	5.1/5.4		
Common Voice—Turkish	16.36	8.53	9.65	34.54	1264
Common Voice—Uzbek	45.69	14.92	16.9	77.51	1355

**Table 2:** Experimental configuration.

Composition	LibriSpeech-100	Common Voice—Turkish	Common Voice—Uzbek
Encoder	VGG/MSPC + 4blstmp	VGG/MSPC + 4blstmp	VGG/MSPC + 4blstmp
Attention	Content-based/location aware/location-lstm	content-based/location-aware/location-lstm	Content-based/Location-aware/Location-lstm
Decoder	Bi-GRU	Bi-GRU	Bi-GRU
CTC_weight_train (λ)	0.2	0.3	0.3
CTC_weight_decode	0.0	0.0	0.0
Adadelta optimizer	*p* = 0.95, ϵ = 10 − 8	*p* = 0.95, ϵ = 10 − 8	*p* = 0.95, ϵ = 10 − 8
RNNLM	-	character	character
Data augmentation	specaugment [56]	specaugment, speed perturb [57], noise	specaugment, speed peturb, noise
LM weight (β)	-	0.5	0.5
Decoding beam size	8	8, 16	8
CTC epoch	10	25	10
Total epoch	20	50	25
Token_type	Character	Character	Character
Vocab_size	30	40	70

**Table 3:** The results of the research were obtained using the test-clean and test-other subsets of LibriSpeech Corpus, showing the CER and WER performance (%) of the SOTA hybrid CTC/attention E2E ASR model with two kinds of attention, three supervised models and our proposed model. Our experimental results are highlighted in bold.

	LibriSpeech-100	CER (%)	WER (%)
Method (No LM)		Test-Clean/Other	Test-Clean/Other
Baseline (location-based) + specaugment		7.64/20.25	16.48/36.69
Baseline (content-based) + specaugment		6.50/18.18	14.69/34.93
Baseline supervised		-/-	14.85/39.95
Semi-supervised [71]		7.6/-	17.5/-
Ensemble (5 models) [72]		-/-	9.62/29.53
Ours (no LM)		**7.2/17.83**	**16.13/33.73**

**Table 4:** The performances comparison of the other two datasets (Turkish and Uzbek) using different methods. Our experimental results are highlighted in bold.

	Common Voice—Turkish	CER (%)	WER (%)
Method			
Baseline (location-based) + specaugment		25.05	55.08
Baseline (content-based) + specaugment		24.76	55.57
Ours (no LM)		24.39	50.91
Ours		**19.23**	**48.01**
	**Common Voice—Uzbek**	**CER (%)**	**WER (%)**
**Method**			
Basline (location-based) + specaugment		12.41	32.31
Basline (content-based) + specaugment		9.23	25.81
Ours (no LM)		8.78	25.78
Ours		**7.96**	**25.23**

**Table 5:** Results of the ablation study of improved attention. Our experimental results are highlighted in bold.

Methods	CER (%)	WER (%)
VGG + BLSTM + BiGRU + location-based attention (specaugment)	25.05	55.08
VGG+BLSTM+BiGRU + content-based attention (specaugment)	24.75	55.57
VGG+BLSTM+BiGRU + location-LSTM attention (specaugment)	**24.53**	**55.22**

**Table 6:** Results of the ablation study of the proposed MSPC architecture. Our experimental results are highlighted in bold.

Methods	CER (%)	WER (%)
MSPC + BLSTM + BiGRU + location-based attention (specaugment)	25.39	53.83
MSPC+BLSTM + BiGRU + content-based attention (specaugment)	24.35	54.61
MSPC+BLSTM + BiGRU + location-LSTM attention (specaugment)	**24.30**	**53.35**

**Table 7:** Results of exploratory experiments with different data augmentation combinations. Here, we do not use LM. Our experimental results are highlighted in bold.

Combination of Data Augmentation	CER (%)	WER (%)
specaugment	24.30	53.35
speed perturbation	23.46	56.11
specaugment + noisy	23.30	51.44
speed perturbation + noisy	**24.39**	**50.91**

**Table 8:** Comparison of experimental results when the beam width is 8 or 16. Our experimental results are highlighted in bold (divided into two cases: with language model and without language model).

Methods (No LM)	Beam Width			
	=8		=16	
	CER	WER	CER	WER
Ours (specaugment)	24.30	53.35	23.42	52.77
Ours (speed perturbation)	23.46	56.11	23.02	54.76
Ours (specaugment + noisy)	23.30	51.44	23.17	50.81
Ours (speed perturbation + noisy)	**24.39**	**50.91**	**19.60**	**48.59**
Ours (speed perturbation + noisy) + LM	**19.23**	**48.01**	**18.97**	**47.49**

## Data Availability

Not applicable.

## References

[1] Li J., Ye G., Das A., Zhao R., Gong Y. Advancing acoustic-to-word CTC model. Proceedings of the 2018 IEEE International Conference on Acoustics, Speech and Signal Processing (ICASSP).

[2] Chang F.-J., Liu J., Radfar M., Mouchtaris A., Omologo M., Rastrow A., Kunzmann S. Context-aware transformer transducer for speech recognition. Proceedings of the 2021 IEEE Automatic Speech Recognition and Understanding Workshop (ASRU).

[3] Chiu C.-C., Sainath T.N., Wu Y., Prabhavalkar R., Nguyen P., Chen Z., Kannan A., Weiss R.J., Rao K., Gonina E. State-of-the-art speech recognition with sequence-to-sequence models. Proceedings of the 2018 IEEE International Conference on Acoustics, Speech and Signal Processing (ICASSP).

[4] Li J., Wu Y., Gaur Y., Wang C., Zhao R., Liu S. (2020). On the comparison of popular end-to-end models for large scale speech recognition. arXiv.

[5] Kim C., Gowda D., Lee D., Kim J., Kumar A., Kim S., Garg A., Han C. A review of on-device fully neural end-to-end automatic speech recognition algorithms. Proceedings of the 2020 54th Asilomar Conference on Signals, Systems, and Computers.

[6] Chan W., Jaitly N., Le Q., Vinyals O. Listen, attend and spell: A neural network for large vocabulary conversational speech recognition. Proceedings of the 2016 IEEE International Conference on Acoustics, Speech and Signal Processing (ICASSP).

[7] Rao K., Sak H., Prabhavalkar R. Exploring architectures, data and units for streaming end-to-end speech recognition with rnn-transducer. Proceedings of the 2017 IEEE Automatic Speech Recognition and Understanding Workshop (ASRU).

[8] Miao Y., Gowayyed M., Metze F. EESEN: End-to-end speech recognition using deep RNN models and WFST-based decoding. Proceedings of the 2015 IEEE Workshop on Automatic Speech Recognition and Understanding (ASRU).

[9] Shewalkar A. (2019). Performance evaluation of deep neural networks applied to speech recognition: RNN, LSTM and GRU. J. Artif. Intell. Soft Comput. Res..

[10] Dong L., Xu S., Xu B. Speech-transformer: A no-recurrence sequence-to-sequence model for speech recognition. Proceedings of the 2018 IEEE International Conference on Acoustics, Speech and Signal Processing (ICASSP).

[11] Watanabe S., Hori T., Kim S., Hershey J.R., Hayashi T. (2017). Hybrid CTC/attention architecture for end-to-end speech recognition. IEEE J. Sel. Top. Signal Process..

[12] Yuan Z., Lyu Z., Li J., Zhou X. (2018). An improved hybrid ctc-attention model for speech recognition. arXiv.

[13] Liu A.H., Lee H.-Y., Lee L.-S. Adversarial training of end-to-end speech recognition using a criticizing language model. Proceedings of the ICASSP 2019-2019 IEEE International Conference on Acoustics, Speech and Signal Processing (ICASSP).

[14] Nakatani T. Improving transformer-based end-to-end speech recognition with connectionist temporal classification and language model integration. Proceedings of the INTERSPEECH 2019.

[15] Simons G.F., Fennig C.D. (2017). Ethnologue: Languages of the World.

[16] Mukhamadiyev A., Khujayarov I., Djuraev O., Cho J. (2022). Automatic Speech Recognition Method Based on Deep Learning Approaches for Uzbek Language. Sensors.

[17] Musaev M., Khujayorov I., Ochilov M. Automatic recognition of Uzbek speech based on integrated neural networks. Proceedings of the World Conference Intelligent System for Industrial Automation.

[18] Thomas S., Seltzer M.L., Church K., Hermansky H. Deep neural network features and semi-supervised training for low resource speech recognition. Proceedings of the 2013 IEEE International Conference on Acoustics, Speech and Signal Processing.

[19] Xu H., Van Hai Do X.X., Xiao X., Chng E. A comparative study of BNF and DNN multilingual training on cross-lingual low-resource speech recognition. Proceedings of the Interspeech.

[20] Dalmia S., Sanabria R., Metze F., Black A.W. Sequence-based multi-lingual low resource speech recognition. Proceedings of the 2018 IEEE International Conference on Acoustics, Speech and Signal Processing (ICASSP).

[21] Diwan A., Vaideeswaran R., Shah S., Singh A., Raghavan S., Khare S., Unni V., Vyas S., Rajpuria A., Yarra C. Mucs 2021: Multilingual and code-switching asr challenges for low resource indian languages. Proceedings of the Annual Conference of the International Speech Communication Association.

[22] Maekawa K. Corpus of Spontaneous Japanese: Its design and evaluation. Proceedings of the ISCA & IEEE Workshop on Spontaneous Speech Processing and Recognition.

[23] Godfrey J.J., Holliman E.C., McDaniel J. SWITCHBOARD: Telephone speech corpus for research and development. Proceedings of the IEEE International Conference on Acoustics, Speech, and Signal Processing.

[24] Bang J.-U., Yun S., Kim S.-H., Choi M.-Y., Lee M.-K., Kim Y.-J., Kim D.-H., Park J., Lee Y.-J., Kim S.-H. (2020). Ksponspeech: Korean spontaneous speech corpus for automatic speech recognition. Appl. Sci..

[25] Kudo T., Richardson J. SentencePiece: A simple and language independent subword tokenizer and detokenizer for Neural Text Processing. Proceedings of the 2018 Conference on Empirical Methods in Natural Language Processing: System Demonstrations.

[26] Ravanelli M., Parcollet T., Plantinga P., Rouhe A., Cornell S., Lugosch L., Subakan C., Dawalatabad N., Heba A., Zhong J. (2021). SpeechBrain: A general-purpose speech toolkit. arXiv.

[27] Kenton J.D.M.-W.C., Toutanova L.K. BERT: Pre-training of Deep Bidirectional Transformers for Language Understanding. Proceedings of the NAACL-HLT.

[28] Hori T., Watanabe S., Zhang Y., Chan W. Advances in Joint CTC-Attention Based End-to-End Speech Recognition with a Deep CNN Encoder and RNN-LM. Proceedings of the INTERSPEECH 2017.

[29] He Y., Sainath T.N., Prabhavalkar R., McGraw I., Alvarez R., Zhao D., Rybach D., Kannan A., Wu Y., Pang R. Streaming end-to-end speech recognition for mobile devices. Proceedings of the ICASSP 2019-2019 IEEE International Conference on Acoustics, Speech and Signal Processing (ICASSP).

[30] Zweig G., Yu C., Droppo J., Stolcke A. Advances in all-neural speech recognition. Proceedings of the 2017 IEEE International Conference on Acoustics, Speech and Signal Processing (ICASSP).

[31] Zeyer A., Beck E., Schlüter R., Ney H. CTC in the context of generalized full-sum HMM training. Proceedings of the Interspeech.

[32] Graves A., Fernández S., Gomez F., Schmidhuber J. Connectionist temporal classification: Labelling unsegmented sequence data with recurrent neural networks. Proceedings of the 23rd International Conference on Machine Learning.

[33] Graves A. (2012). Sequence transduction with recurrent neural networks. arXiv.

[34] Das A., Li J., Zhao R., Gong Y. Advancing connectionist temporal classification with attention modeling. Proceedings of the 2018 IEEE International Conference on Acoustics, Speech and Signal Processing (ICASSP).

[35] Chiu C.-C., Han W., Zhang Y., Pang R., Kishchenko S., Nguyen P., Narayanan A., Liao H., Zhang S., Kannan A. A comparison of end-to-end models for long-form speech recognition. Proceedings of the 2019 IEEE Automatic Speech Recognition and Understanding Workshop (ASRU).

[36] Kim S., Hori T., Watanabe S. Joint CTC-attention based end-to-end speech recognition using multi-task learning. Proceedings of the 2017 IEEE International Conference on Acoustics, Speech and Signal Processing (ICASSP).

[37] Bahdanau D., Cho K.H., Bengio Y. Neural machine translation by jointly learning to align and translate. Proceedings of the 3rd International Conference on Learning Representations.

[38] Chorowski J.K., Bahdanau D., Serdyuk D., Cho K., Bengio Y. Attention-based models for speech recognition. Proceedings of the Advances in Neural Information Processing Systems.

[39] Vaswani A., Shazeer N., Parmar N., Uszkoreit J., Jones L., Gomez A.N., Kaiser Ł., Polosukhin I. Attention is all you need. Proceedings of the Advances in Neural Information Processing Systems.

[40] Hori T., Watanabe S., Hershey J.R. Joint CTC/attention decoding for end-to-end speech recognition. Proceedings of the 55th Annual Meeting of the Association for Computational Linguistics.

[41] Seki H., Hori T., Watanabe S., Moritz N., Le Roux J. Vectorized Beam Search for CTC-Attention-Based Speech Recognition. Proceedings of the INTERSPEECH.

[42] Moritz N., Hori T., Le Roux J. Triggered attention for end-to-end speech recognition. Proceedings of the ICASSP 2019—2019 IEEE International Conference on Acoustics, Speech and Signal Processing (ICASSP).

[43] Wu L., Li T., Wang L., Yan Y. (2019). Improving hybrid CTC/Attention architecture with time-restricted self-attention CTC for end-to-end speech recognition. Appl. Sci..

[44] Radford A., Wu J., Child R., Luan D., Amodei D., Sutskever I. (2019). Language models are unsupervised multitask learners. OpenAI Blog.

[45] Sanh V., Debut L., Chaumond J., Wolf T. (2019). DistilBERT, a distilled version of BERT: Smaller, faster, cheaper and lighter. arXiv.

[46] Deng K., Cheng G., Miao H., Zhang P., Yan Y. History utterance embedding transformer lm for speech recognition. Proceedings of the ICASSP 2021—2021 IEEE International Conference on Acoustics, Speech and Signal Processing (ICASSP).

[47] Kannan A., Wu Y., Nguyen P., Sainath T.N., Chen Z., Prabhavalkar R. An analysis of incorporating an external language model into a sequence-to-sequence model. Proceedings of the 2018 IEEE International Conference on Acoustics, Speech and Signal Processing (ICASSP).

[48] Sriram A., Jun H., Satheesh S., Coates A. Cold Fusion: Training Seq2Seq Models Together with Language Models. Proceedings of the INTERSPEECH 2018.

[49] Yu F.-H., Chen K.-Y. (2021). Non-autoregressive transformer-based end-to-end ASR using BERT. arXiv.

[50] Deng K., Cao S., Zhang Y., Ma L. Improving hybrid ctc/attention end-to-end speech recognition with pretrained acoustic and language models. Proceedings of the 2021 IEEE Automatic Speech Recognition and Understanding Workshop (ASRU).

[51] Baevski A., Zhou Y., Mohamed A., Auli M. wav2vec 2.0: A framework for self-supervised learning of speech representations. Proceedings of the Advances in Neural Information Processing Systems.

[52] Mamyrbayev O., Alimhan K., Zhumazhanov B., Turdalykyzy T., Gusmanova F. End-to-end speech recognition in agglutinative languages. Proceedings of the Asian Conference on Intelligent Information and Database Systems.

[53] Yi C., Zhou S., Xu B. (2021). Efficiently fusing pretrained acoustic and linguistic encoders for low-resource speech recognition. IEEE Signal Process. Lett..

[54] Mamyrbayev O.Z., Oralbekova D.O., Alimhan K., Nuranbayeva B.M. (2022). Hybrid end-to-end model for Kazakh speech recognition. Int. J. Speech Technol..

[55] Yu C., Kang M., Chen Y., Wu J., Zhao X. (2020). Acoustic modeling based on deep learning for low-resource speech recognition: An overview. IEEE Access.

[56] Park D.S., Chan W., Zhang Y., Chiu C.-C., Zoph B., Cubuk E.D., Le Q.V. SpecAugment: A Simple Data Augmentation Method for Automatic Speech Recognition. Proceedings of the INTERSPEECH 2019.

[57] Ko T., Peddinti V., Povey D., Khudanpur S. Audio augmentation for speech recognition. Proceedings of the Sixteenth Annual Conference of the International Speech Communication Association.

[58] Hasija T., Kadyan V., Guleria K., Alharbi A., Alyami H., Goyal N. (2022). Prosodic Feature-Based Discriminatively Trained Low Resource Speech Recognition System. Sustainability.

[59] Singh T.P., Gupta S., Garg M., Gupta D., Alharbi A., Alyami H., Anand D., Ortega-Mansilla A., Goyal N. (2022). Visualization of Customized Convolutional Neural Network for Natural Language Recognition. Sensors.

[60] Cho J., Baskar M.K., Li R., Wiesner M., Mallidi S.H., Yalta N., Karafiat M., Watanabe S., Hori T. Multilingual sequence-to-sequence speech recognition: Architecture, transfer learning, and language modeling. Proceedings of the 2018 IEEE Spoken Language Technology Workshop (SLT).

[61] Yi J., Tao J., Wen Z., Bai Y. Adversarial multilingual training for low-resource speech recognition. Proceedings of the 2018 IEEE International Conference on Acoustics, Speech and Signal Processing (ICASSP).

[62] Liang S., Yan W. (2022). Multilingual speech recognition based on the end-to-end framework. Multimed. Tools Appl..

[63] Hannun A. (2017). Sequence modeling with ctc. Distill.

[64] Li J. (2022). Recent advances in end-to-end automatic speech recognition. APSIPA Trans. Signal Inf. Process..

[65] Chorowski J., Jaitly N. Towards Better Decoding and Language Model Integration in Sequence to Sequence Models. Proceedings of the INTERSPEECH 2017.

[66] Ardila R., Branson M., Davis K., Kohler M., Meyer J., Henretty M., Morais R., Saunders L., Tyers F., Weber G. Common Voice: A Massively-Multilingual Speech Corpus. Proceedings of the 12th Language Resources and Evaluation Conference.

[67] Panayotov V., Chen G., Povey D., Khudanpur S. Librispeech: An asr corpus based on public domain audio books. Proceedings of the 2015 IEEE international conference on acoustics, speech and signal processing (ICASSP).

[68] Simonyan K., Zisserman A. (2014). Very deep convolutional networks for large-scale image recognition. arXiv.

[69] Snyder D., Chen G., Povey D. (2015). Musan: A music, speech, and noise corpus. arXiv.

[70] Ko T., Peddinti V., Povey D., Seltzer M.L., Khudanpur S. A study on data augmentation of reverberant speech for robust speech recognition. Proceedings of the 2017 IEEE International Conference on Acoustics, Speech and Signal Processing (ICASSP).

[71] Baskar M.K., Watanabe S., Astudillo R.F., Hori T., Burget L., Černocký J. Semi-supervised sequence-to-sequence ASR using unpaired speech and text. Proceedings of the Annual Conference of the International Speech Communication Association.

[72] Kahn J., Lee A., Hannun A. Self-training for end-to-end speech recognition. Proceedings of the ICASSP 2020-2020 IEEE International Conference on Acoustics, Speech and Signal Processing (ICASSP).

